# Colonization, Infection, and the Accessory Genome of *Klebsiella pneumoniae*

**DOI:** 10.3389/fcimb.2018.00004

**Published:** 2018-01-22

**Authors:** Rebekah M. Martin, Michael A. Bachman

**Affiliations:** Department of Pathology, Michigan Medicine, University of Michigan, Ann Arbor, MI, United States

**Keywords:** *Klebsiella pneumoniae*, antibiotic resistance, carbapenem resistance, hypervirulent, colonization, hospital-acquired infection, CRE

## Abstract

*Klebsiella pneumoniae* is a Gram-negative pathogen that has a large accessory genome of plasmids and chromosomal gene loci. This accessory genome divides *K. pneumoniae* strains into opportunistic, hypervirulent, and multidrug-resistant groups and separates *K. pneumoniae* from two closely related species, *Klebsiella variicola* and *Klebsiella quasipneumoniae*. Some strains of *K. pneumoniae* act as opportunistic pathogens, infecting critically ill and immunocompromised patients. These *K. pneumoniae* are a common cause of health-care associated infections including pneumonia, urinary tract infections (UTIs), and bloodstream infections. *K. variicola* and *K. quasipneumoniae* are often clinically indistinguishable from opportunistic *K. pneumoniae*. Other strains of *K. pneumoniae* are hypervirulent, infecting healthy people in community settings and causing severe infections including pyogenic liver abscess, endophthalmitis, and meningitis. A third group of *K. pneumoniae* encode carbapenemases, making them highly antibiotic-resistant. These strains act as opportunists but are exceedingly difficult to treat. All of these groups of *K. pneumoniae* and related species can colonize the gastrointestinal tract, and the accessory genome may determine if a colonizing strain remains asymptomatic or progresses to cause disease. This review will explore the associations between colonization and infection with opportunistic, antibiotic-resistant, and hypervirulent *K. pneumoniae* strains and the role of the accessory genome in distinguishing these groups and related species. As *K. pneumoniae* infections become progressively more difficult to treat in the face of antibiotic resistance and hypervirulent strains, an increased understanding of the epidemiology and pathogenesis of these bacteria is vital.

## Introduction

*Klebsiella pneumoniae* was first described by Carl Friedlander in 1882 as a bacterium isolated from the lungs of patients who had died from pneumonia (Friedlaender, 1882). *Klebsiella* species are found ubiquitously in nature, including in plants, animals, and humans. They are the causative agent of several types of infections in humans, including respiratory tract infections, urinary tract infections (UTIs), and bloodstream infections (Podschun and Ullmann, 1998). Classically, these infections occur in hospitalized or otherwise immunocompromised patients and are routinely treated with β-lactams and other antibiotics effective against *Enterobacteriaceae* (Mandell, 2005). However, antibiotic-resistant *K. pneumoniae* and hypervirulent *K. pneumoniae* strains have emerged separately across the world. Furthermore, recent advances in molecular capabilities have shown that a portion of clinical isolates identified as *K. pneumoniae* are in fact other *Klebsiella* species (Brisse and Verhoef, 2001; Brisse et al., 2004; Maatallah et al., 2014; Berry et al., 2015; Long et al., 2017a). This review will focus on the epidemiology of endemic opportunistic, epidemic antibiotic-resistant, and emerging hypervirulent strains of *K. pneumoniae*, and the role of the accessory genome in each pathotype. Understanding how these emerging strains and species are similar and how they differ from one another, as well as the genetic factors contributing to their epidemiology, is necessary to successfully combat these infections.

## *Klebsiella pneumoniae*: an opportunistic, hospital-acquired infection

*Klebsiella pneumoniae* is a Gram-negative pathogenic bacterium. On agar media, it has a mucoid phenotype that is conferred by the polysaccharide capsule attached to the bacterial outer membrane, and ferments lactose. *K. pneumoniae* is part of the *Enterobacteriaceae* family, which is comprised of other familiar pathogens such as *Escherichia coli, Yersinia* species, *Salmonella* species, and *Shigella* species. *K. pneumoniae*, a leading cause of hospital-acquired infections (HAIs) in the United States (Magill et al., 2014), has classically been considered an opportunistic pathogen, since it typically causes infections in hospitalized or otherwise immunocompromised individuals (Podschun and Ullmann, 1998). As the virulence of these bacteria and the demographic features of the patients they infect begin to shift, understanding how *K. pneumoniae* is transmitted and the factors responsible for pathogenicity is important in treating infected patients.

*Klebsiella* species have been identified as the third leading cause of HAIs in the United States (9.9%) behind *Clostridium difficile* and *Staphylococcus aureus* (Magill et al., 2014). *K. pneumoniae* causes serious infections including pneumonia, UTIs, and bloodstream infections (Magill et al., 2014). In fact, *Klebsiella* species have been identified as the number three cause of HA pneumonia in the United States, defined as a pneumonia occurring ≥48 h after hospital admission (Magill et al., 2014). *Klebsiella* species are also a leading cause of ventilator-associated pneumonia (VAP) among patients in intensive care units (ICUs) (Kalanuria et al., 2014; Selina et al., 2014), and VAP is responsible for 83% of hospital-acquired (HA) pneumonias (Richards et al., 2000). Mortality rates in *K. pneumoniae* pneumonia have been reported as high as 50% (Podschun and Ullmann, 1998).

*K. pneumoniae* are the second leading cause of bloodstream infections (BSI) caused by Gram-negative bacteria, behind only *E. coli* (Podschun and Ullmann, 1998; Magill et al., 2014). Cancer is the primary underlying disease associated with hospital-acquired BSI, while liver disease and diabetes mellitus had the highest association among community-acquired (CA) *K. pneumoniae* BSI (Kang et al., 2006). BSI can be a primary infection with no identifiable source. However, BSI is often a secondary infection that results from dissemination into the bloodstream from a known source. Common sources of secondary BSI include the urinary tract, the gastrointestinal tract, intravenous or urinary catheters, and respiratory sites (Montgomerie and Ota, 1980). The case mortality rate of BSI due to *K. pneumoniae* is ~20–30%, and the population mortality rate is estimated at 1.3 per 100,000 people (Podschun and Ullmann, 1998; Meatherall et al., 2009).

The urinary tract is the most common site of infection by *K. pneumoniae* (Podschun and Ullmann, 1998). As with other infections, UTI due to *K. pneumoniae* are associated with diabetes mellitus (Lye et al., 1992). Catheter-associated UTIs (CAUTIs) are another infection caused by *K. pneumoniae*. It is thought that these are facilitated by the ability for form biofilms and adhere to catheters (Schroll et al., 2010). *Klebsiella* are also responsible for wound/surgical site infections. This site represents ~13% of all infections caused by *Klebsiella* (Podschun and Ullmann, 1998; Magill et al., 2014). Together, *K. pneumoniae* infections at each of these body sites represent an endemic opportunistic pathogen that is a substantial burden for healthcare.

### *Klebsiella pneumoniae* commonly colonize human mucosal surfaces

The environment likely acts as a reservoir for human acquisition of *K. pneumoniae*, either as colonization or an infection. *K. pneumoniae* is frequently found in water, sewage, soil, and plant surfaces (Bagley, 1985; Podschun et al., 2001). Several studies have shown that *K. pneumoniae* in the environment are very similar to their clinical counterparts in biochemical patterns, virulence and pathogenicity, and bacteriocin susceptibility patterns (Matsen et al., 1974; Podschun et al., 1992; Podschun and Ullmann, 1996; Struve and Krogfelt, 2004), though capsule type representation differs between clinical/fecal and environmental sources (Podschun, 1990). However, environmental *K. pneumoniae* are significantly more susceptible to antibiotics than clinical *K. pneumoniae* (Matsen et al., 1974), suggesting that selective pressure exists in a clinical setting. Interestingly, both environmental temperature and dewpoint are positively predictive of increased bloodstream infection caused by *K. pneumoniae*, suggesting that acquisition from the environment may vary with season and climate (Anderson et al., 2008). In the hospital, there are several potential sources of transmission of *K. pneumoniae*. One source of transmission is person-to-person contact between healthcare workers and patients, with healthcare workers' hands being a significant source (Casewell and Phillips, 1977; Jarvis et al., 1985). Contaminated surfaces and instrumentation have also been identified as sources of transmission (Jarvis et al., 1985).

Once acquired, *K. pneumoniae* colonizes the mucosal surfaces in humans, including the nasopharynx and the gastrointestinal tract (Podschun and Ullmann, 1998). These bacteria can be found on skin, but are considered transient at this site rather than colonizing (Kloos and Musselwhite, 1975). Colonization rates differ by body site and whether *K. pneumoniae* are community- or hospital-acquired. Specifically, rates of CA colonization of the nasopharynx have been reported from 3 to 15% (Davis and Matsen, 1974; Wolf et al., 2001; Farida et al., 2013; Dao et al., 2014), and are typically higher in adults than children (Wolf et al., 2001; Farida et al., 2013). Nasopharyngeal colonization has also been associated with alcohol consumption (Fuxench-Lopez and Ramirez-Ronda, 1978; Dao et al., 2014). HA nasopharyngeal colonization rates are slightly higher, up to 19% (Pollack et al., 1972). In contrast to the nasopharynx, rates of CA gastrointestinal colonization vary but can reach as high as 35% (Davis and Matsen, 1974). Furthermore, rates of gastrointestinal colonization increase among hospitalized patients and have been reported as high as 77% (Podschun and Ullmann, 1998), though recent studies demonstrate rates around 20% (Martin et al., 2016; Gorrie et al., 2017). The wide variation in colonization rates may be due to differences in the patient populations sampled several decades ago (Rose and Schreier, 1968; Thom, 1970; Selden et al., 1971; Pollack et al., 1972). Colonization rates also increase following antibiotic treatment (Rose and Schreier, 1968). Among body sites, gastrointestinal colonization is likely a common and significant reservoir in terms of risk of transmission and infection (Dorman and Short, 2017). Published colonization rates are based on detectable colonization by nasopharyngeal, rectal, or fecal sampling. It is possible that many more people are truly colonized, but that colonization is only detected by culture when the density exceeds a certain threshold.

### Progression from colonization to infection

Decades ago, serotyping based on the *Klebsiella* capsule indicated that gastrointestinal colonization is an important reservoir for strains causing healthcare-associated infections (Selden et al., 1971; Montgomerie, 1979). However, serotyping does not provide sufficient resolution to distinguish closely related strains. In seeking to understand how infection with *K. pneumoniae* progresses, two recent studies have investigated the link between colonization with *K. pneumoniae* and subsequent infection (Martin et al., 2016; Gorrie et al., 2017). Gastrointestinal carriage of *K. pneumoniae* was significantly associated with subsequent infections in hospitalized patients (Odds ratio > 4), even after adjusting for other risk factors for infection, and ~5% of colonized patients went on to get an infection. Both studies used genomic methods to explore if infections are caused by patients' colonizing strains and each found ~80% concordance between infecting and colonizing isolates of *K. pneumoniae* within infected patients. Understanding colonization as an important step in progression to infection provides the rationale for identifying colonized patients and potentially establishing intervention protocols to prevent subsequent infection.

If colonization is a step in progression to infection, then understanding the mechanisms of that progression is important. A 1990 study noted that the capsule serotypes of *Klebsiella* clinical isolates were more similar to fecal sample serotypes than those of environmental samples, suggesting that colonization and infection may be linked (Podschun, 1990). Several studies have suggested that the intestinal tract is a reservoir for hospital-acquired pathogens and have further suggested mechanisms for dissemination, however most have focused on pathogens other than *K. pneumoniae* (Donskey, 2004). Though the mechanism of progression from intestinal *K. pneumoniae* colonization to infection is not clearly understood, there are some apparent risk factors. Intestinal domination by Proteobacteria, which taxonomically includes *K. pneumoniae*, leads to a five-fold increase in the risk of bacteremia in allogenic hematopoietic stem cell transplant patients (Taur et al., 2012), suggesting that bacterial density of colonizing strains plays a role in progression to disease. Procedures such as endoscopy are a potential further source of endogenous infection (Spach et al., 1993). Several underlying diseases have also been identified as risk factors for infection, since they weaken host defenses and therefore increase susceptibility to infection. Specifically, cancer, diabetes mellitus, and alcoholism are associated with both CA and HA *K. pneumoniae* infection (Tsay et al., 2002; Happel and Nelson, 2005; Tsai et al., 2010), though whether they are associated with progression from colonization or only with infection is unclear. Compared to intestinal colonization, fluid and electrolyte disorders, neurologic disorders, and prior hospital admissions have been identified as significantly and independently associated with infection in hospitalized patients (Martin et al., 2016). While various risk factors for infection with *K. pneumoniae* have been identified, risk factors specifically associated with progression from colonization to infection have not been elucidated. Further studies are needed to understand how this progression occurs and what risk factors may be involved. In addition to epidemiological risk factors, variations in the accessory genome may modulate the risk for progression to disease.

### The accessory genome

Within a bacterial species, there is typically a set of genes that is conserved amongst all members. This set of genes is considered the core genome. In *K. pneumoniae* the core genome, defined as present in >95% of isolates, is currently estimated to be comprised of ~2,000 genes (Holt et al., 2015). Genes that vary between isolates are referred to as the accessory genome. This includes chromosomally encoded genes and genes on plasmids. Since *K. pneumoniae* genomes are typically between 5,000 and 6,000 genes, this means that the majority of the genome is comprised of accessory genes. Genes in the accessory genome can aid in specific processes, such as nitrogen fixation (Fouts et al., 2008). They can also encode specific virulence factors, as discussed below. The accessory genome also carries genes encoding various antibiotic-resistant enzymes and mechanisms (Bi et al., 2015). Accessory genes can be acquired due to horizontal gene transfer among bacterial species, as evidenced by the presence of genomic islands and mobile genetic elements in many isolates. The genes encoded in genomic islands can help isolates adapt to specific sites of infection or colonization (Chen et al., 2010; Zhang et al., 2011; van Aartsen et al., 2012). Elements of the accessory genome can be identified or predicted using various *in silico* applications, including computation of GC content and comparative genomic analysis (Ou et al., 2007; Zhang et al., 2014). A recent study of 328 *Klebsiella* isolates identified almost 30,000 unique protein-coding sequences (Holt et al., 2015), estimating the current known *Klebsiella* “pangenome.” The authors further demonstrated that the pangenome is open, indicating that there are more accessory genes yet to be identified and characterized.

Not all *K. pneumoniae* strains cause disease in animal models of infection (Fouts et al., 2008; Fodah et al., 2014). Similarly, not all colonizing strains go on to cause disease in humans (Martin et al., 2016). In fact, these bacteria are traditionally considered commensals and opportunistic pathogens (Lau et al., 2008). Of those that do cause disease, certain genes, operons, and the high pathogenicity island encoding yersiniabactin (discussed below) have been identified in *K. pneumoniae* that are associated with virulence (Lau et al., 2007; Lawlor et al., 2007; Fodah et al., 2014; Holt et al., 2015). This indicates that progression to disease is not only reliant on host immunocompetence, but also on genes the bacteria possess.

### The accessory genome in progression to disease and virulence

*K. pneumoniae* virulence factors are encoded by genes in both the core and accessory genomes. Established virulence factors in *K. pneumoniae* include capsule, lipopolysaccharide, siderophores, and pili (Podschun and Ullmann, 1998). Allantoin utilization, other iron uptake systems, efflux pumps, and a type VI secretion system have been identified as virulence factors more recently. Allantoin utilization will be discussed with hypervirulent strains. These genes may determine the severity of an infection, and therefore the virulence of the infecting strain. They may also determine the ability of a colonizing strain to progress to infection, defining the pathogenic potential of a given strain.

The polysaccharide capsule is one of the most important virulence factors used by *K. pneumoniae*. It is primarily used to assist in evading the immune system during infection, by protecting bacteria from opsonophagocytosis (Domenico et al., 1994) and serum killing (Merino et al., 1992). The capsule is generated by the capsular polysaccharide synthesis (*cps*) locus in *K. pneumoniae*, and is a structure that lies on the outside of the bacterial cell attached to the outer membrane. It is composed of repeating subunits of four to six sugars, as well as uronic acids (Podschun and Ullmann, 1998). Based on serological testing, 77 capsular types have been identified (Ørskov and Ørskov, 1984). Recent advances in molecular techniques have led to further discrimination between *K. pneumoniae* types as well as complete sequencing of the *cps* locus in all serotypes (Pan et al., 2015). An example of increased discrimination is seen in *wzi* typing, which identifies types based on allelic differences in the *wzi* gene of the *cps* locus (Brisse et al., 2013). This method initially identified 135 distinct *wzi* types that correspond with traditional serological K types. Many of these genes are conserved, but other *cps* genes and gene alleles are present or absent depending on the capsule type. Thus, capsular synthesis is carried out by a combination of core and accessory genes.

Lipopolysaccharide (LPS), also termed endotoxin, is a major component decorating the outer membrane of Gram-negative bacteria. LPS is widely recognized as the most powerful mediator of septic shock caused by bacteria. Host sensing of LPS via Toll-like receptor 4 (TLR4) leads to an inflammatory cascade (Roger et al., 2009). It is this host response, rather than LPS itself, that leads to the devastating pathogenesis of sepsis and septic shock. LPS molecules are comprised of lipid A, a core domain, and the O-antigen. Variations in O-antigen structures provide various O-serotypes. In *K. pneumoniae* there are nine main O-serotypes. Three of these, O1, O2, and O3, are responsible for almost 80% of all *Klebsiella* infections (Trautmann et al., 1997; Hansen et al., 1999; Follador et al., 2016). *K. pneumoniae* isolates with shortened or absent O-antigen (rough LPS) are sensitive to serum complement, while isolates with full-length O-antigen (smooth LPS) are resistant to serum complement (Merino et al., 1992). Variations in LPS can also play a role in protecting bacteria from antimicrobial peptides, including polymyxin antibiotics (Papo and Shai, 2005; Cheng et al., 2015). As a primary component of the outer membrane of *K. pneumoniae*, LPS is considered part of the core genome.

Siderophores are high affinity, low-molecular weight iron-chelating molecules secreted by various bacteria to aid in iron acquisition (Griffiths, 1988). *K. pneumoniae* secrete multiple types of siderophores (Holden and Bachman, 2015). One of the common catecholates secreted by *K. pneumoniae* is enterobactin (Ent), which is encoded in the *K. pneumoniae* core genome (O'Brien and Gibson, 1970; Pollack and Neilands, 1970). Since Ent is a common siderophore, the innate immune system has developed a way to bind Ent, preventing bacteria from acquiring iron (Smith, 2007). Bacteria have therefore developed other siderophores, which are encoded in the accessory genome, to counteract this. A second catecholate siderophore is a glucosylated Ent-derivative, salmochelin (Sal). Sal, is a mechanism to evade the innate immune system (Hantke et al., 2003). Mixed-type siderophores such as yersiniabactin (Ybt) and aerobactin (Aer) are also commonly secreted by *K. pneumoniae*. The various siderophores are associated with different sites and severity of infection, and their pathogenic functions extend beyond simply iron acquisition. Ybt was first identified in *Yersinia* species, and the genes encoding the biosynthesis, transport, and regulation of this molecule are located on a transposable chromosomal fragment termed a “high pathogenicity island” (Carniel, 2001). Secretion and utilization of Ybt is associated with respiratory tract infections in patients and is sufficient to promote pneumonia in a murine model (Bachman et al., 2011). World-wide, Ybt is the most common virulence factor associated with human *K. pneumoniae* infections (Holt et al., 2015). Aer has also been identified as a major virulence factor in *K. pneumoniae* infections. Aer is typically plasmid-encoded (Nassif and Sansonetti, 1986). In addition to providing iron for *K. pneumoniae* replication, secretion of siderophores by *K. pneumoniae* isolates also induces inflammation and bacterial dissemination. Siderophore-dependent progression from pneumonia to bacteremia requires the host transcriptional regulatory protein HIF-1α that is activated by iron chelation (Holden et al., 2016). Siderophore systems are frequently dependent on TonB, a protein that regulates the transport of iron across the bacterial outer membrane (Moeck and Coulton, 1998). In fact, TonB itself is considered a virulence factor in *K. pneumoniae* (Hsieh et al., 2008). TonB is encoded by a gene on the bacterial chromosome. It is conserved among *K. pneumoniae*, and is used as one of the housekeeping genes for multilocus sequence typing in these bacteria (Diancourt et al., 2005). Whereas Ent and TonB are part of the core genome, Sal, Ybt and Aer are part of the accessory genome.

A critical step in progression to infection is for bacteria to adhere to host surfaces. In *K. pneumoniae*, this is frequently achieved using pili (fimbriae). Pili are filamentous structures extending from the surface of bacteria. They can be as long as 10 μm and between 1-11 nm in diameter (Ofek and Doyle, 1994). There are two common types of pili found on *K. pneumoniae*: type 1 (*fim*) pili and type 3 (*mrk*) pili. Type 1 pili are thought to aid virulence through their ability to adhere to human mucosal or epithelial surfaces. Type 3 pili similarly adhere to cell surfaces, but importantly have been identified as strong promoters of biofilm formation (Schroll et al., 2010). Both *fim* and *mrk* pili are considered part of the core genome (Andrade et al., 2014; Lery et al., 2014). It is thought that both types of pili play a role in colonization of urinary catheters, leading to catheter-associated UTIs (Murphy et al., 2013). In addition to *fim* and *mrk* pili, a number of additional usher-type pili have been identified in *K. pneumoniae*, with an average of ~8 pili clusters per strain (Wu et al., 2010; Khater et al., 2015). Based on varying gene frequencies, some of these appear to be part of the accessory genome.

Efflux pumps have frequently been associated with antibiotic resistance in *K. pneumoniae* due to their ability to export antibiotics from bacterial cells (Filgona et al., 2015). Interestingly, the efflux pump AcrAB contributes to virulence in murine respiratory infections caused by *K. pneumoniae* (Padilla et al., 2010). In other bacteria, efflux pumps have been demonstrated to mediate resistance against host-derived antimicrobial peptides, which are an important aspect of the host innate immune system (Shafer et al., 1998; Bengoechea and Skurnik, 2000; Tzeng et al., 2005; Pamp et al., 2008). It is possible that this mechanism plays a similar role in the virulence of *K. pneumoniae*.

The type VI secretion system (T6SS), first identified in *V. cholerae* (Pukatzki et al., 2006), is a syringe-like apparatus anchored within the bacterial cell membrane that serves to inject various effector molecules and toxins into other cells (Journet and Cascales, 2016). Present in several Gram-negative species, the T6SS has been shown to target both other bacteria as well as eukaryotic cells, suggesting a dual role in both competition and pathogenesis. Bioinformatic analysis of *K. pneumoniae* genomes has identified the presence of putative T6SS gene clusters, with up to 3 loci per strain, although the number and gene content vary (Sarris et al., 2011). More recent studies have begun to characterize T6SS effectors in *K. pneumoniae*. In hypervirulent strain Kp52.145, the phospholipase PLD1 is required for virulence in a pneumonia model, and in the carbapenemase producing strain HS11286 the phospholipase Tle1^KP^ promotes inter- and intra-species killing and antibiotics induce its secretion (Lery et al., 2014; Liu et al., 2017). T6SS secretion systems appear to be present in both the core and accessory genome, promote competition with other bacteria in sites of colonization, and enhance fitness in sites of infection.

## Emergence of antibiotic resistance in *Klebsiella pneumoniae*

The Centers for Disease Control and Prevention estimates that more than two million people contract infections due to antibiotic-resistant microorganisms each year in the United States. Of those infected, ~23,000 die (CDC, 2014). There are multiple factors believed to contribute to the spread of antibiotic resistance, including inappropriate antibiotic use in healthcare and agriculture, and lack of new antimicrobial therapeutics (CDC, 2014). *K. pneumoniae* are one of several bacteria that have experienced a dramatic increase in antibiotic resistance in the past decades. Several mechanisms of antibiotic resistance are found in *K. pneumoniae*, with resistance to β-lactams having the greatest impact on effective treatment. Colonization with antibiotic-resistant *K. pneumoniae* has been associated with subsequent infection with antibiotic-resistant *K. pneumoniae* in hospitalized patients, although the progression from colonization to infection is incompletely understood. The accessory genome is central to antibiotic resistance in *K. pneumoniae*, with plasmid based resistance genes rapidly reducing the armamentarium of effective antibiotics against this pathogen.

### β-Lactamase-producing *Klebsiella pneumoniae*

Resistance of bacteria to β-lactam antibiotics emerged before penicillin was widely used to treat infections. Alexander Fleming was the first to note that *E. coli* and other bacteria were not inhibited by penicillin (Fleming, 1929), a resistance which was later attributed to an enzyme produced by these bacteria (Abraham and Chain, 1940). Resistance to β-lactam antibiotics is achieved through hydrolysis of the antibiotic β-lactam ring by β-lactamases. In *K. pneumoniae*, resistance to some β-lactams is intrinsic since the enzyme is encoded in the core genome of the species. For example, SHV is consistently found in the chromosome, and corresponding ampicillin resistance is a hallmark of the species (Babini and Livermore, 2000; Bialek-Davenet et al., 2014). Other β-lactamases are part of the accessory genome. In the 1960s the first plasmid-mediated β-lactamase, TEM-1, was discovered in *E. coli* (Datta and Kontomichalou, 1965). *K. pneumoniae* are also known to harbor plasmid-mediated β-lactamases, such as AmpC enzymes which confer resistance to most penicillin antibiotics (Jacoby, 2009). β-lactamase enzymes are thought to have evolved from penicillin binding proteins due to selective pressure in the environment (Medeiros, 1984; Kelly et al., 1986; Massova and Mobashery, 1998; Meroueh et al., 2003).

### Extended-spectrum β-lactamases

Extended-spectrum β-lactamases (ESBL) are plasmid based resistance mechanisms that are part of the accessory genome. ESBL-producing *K. pneumoniae* were first identified in Europe in 1983 (Knothe et al., 1983) and in the United States in 1989 (Quinn et al., 1989). ESBLs are able to hydrolyze oxyimino-cephalosporins, such as third-generation cephalosporins and aztreonam, but are inhibited by clavulanic acid (Bush et al., 1995). Frequently plasmids encoding β-lactamases also possess resistance genes to other antibiotics as well as heavy metals (Jacoby and Sutton, 1991). Therefore, ESBLs are tightly linked to other accessory genes that could improve fitness of the strain it is in. And plasmid-based resistance mechanisms are associated with certain lineages that may have a characteristic accessory genome. For instance, a recent study identified that *K. pneumoniae* clonal group 307 (CG307) is associated with ESBL infections (Long et al., 2017b). Carbapenems have typically been the drug of choice to treat severe infections caused by ESBL-producing bacteria.

### Carbapenem-resistant *Klebsiella pneumoniae* (CR-Kp)

Perhaps due to the selective pressure of treating ESBL infections with carbapenems, carbapenem resistance has emerged and *K. pneumoniae* is the most common carbapenem-resistant *Enterobacteriaceae* (CRE). In 2013 the CDC declared CRE an urgent threat to public health in the United States (CDC, 2014). Of the ~9,000 infections due to CRE, *Klebsiella* species are responsible for ~80% of infections (CDC, 2014).

Carbapenem resistance is primarily driven by the accessory genome, sometimes in combination with mutations in the core genome. Carbapenem resistance in *K. pneumoniae* can be mediated in part through up-regulation of efflux pumps (Filgona et al., 2015) and alteration of outer membrane porins in the core genome (Kaczmarek et al., 2006), and hyperproduction of ESBL enzymes or AmpC β-lactamases in the accessory genome (Bush and Jacoby, 2010). For instance, hyperproduction of an ESBL or AmpC enzyme combined with a porin mutation can lead to a resistance phenotype, particularly to ertapenem (Bradford et al., 1997; García-Fernández et al., 2010). The most worrisome mechanism of carbapenem resistance is through plasmid-mediated carbapenemases. *K. pneumoniae* carbapenemase (KPC) β-lactamases are class A serine carbapenemases and the most frequent carbapenemases found in *K. pneumoniae*. KPC carbapenemases are associated with clonal group 258 (CG258) (Samuelsen et al., 2009; Breurec et al., 2013). Prominent sequence types within CG258 include ST258 that is found in Europe and North and South America, and ST11 in Asia (Baraniak et al., 2009; Kitchel et al., 2009; García-Fernández et al., 2012; Liu et al., 2012; Nicoletti et al., 2012). As of 2015, 22 KPC variants have been identified worldwide according to the Lahey Clinic website (http://www.lahey.org/studies/other.asp#table1). The association between the KPC gene and certain clonal groups suggests that these resistance plasmids have become stable members of the accessory genome in certain *K. pneumoniae* lineages.

Additional types of carbapenemases have also emerged in the *K. pneumoniae* accessory genome. The New Delhi metallo-β-lactamase-1 (NDM-1) is a plasmid-encoded class B metallo-β-lactamase (MBL). MBLs are characterized by a requirement for zinc at their active site and infections with MBL-producing strains are frequently associated with travel to and hospitalization in endemic regions (van der Bij and Pitout, 2012). For example, NDM-1 was discovered in a *K. pneumoniae* clinical urinary culture isolate from a Swedish patient who had recently traveled to India (Yong et al., 2009). Since then, acquisition of NDM-1 isolates has been strongly associated with individuals of Indian descent who travel to or live in the Indian subcontinent (Yong et al., 2009; Kumarasamy et al., 2010; Chen et al., 2017). *K. pneumoniae* carrying the Verona-integron encoded metallo-β-lactamase carbapenemases (VIM) were first detected in the United States in 2010 (Centers for Disease and Prevention, 2010). VIM-encoding isolates are endemic to Greece and Italy, and infections in the United States are associated with travel to and been hospitalization in these countries (Lauretti et al., 1999; van der Bij and Pitout, 2012; Lascols et al., 2013). VIM variants (*bla*_VIM_) are carried on integrons that can be integrated into either the chromosome or carried on plasmids (Lauretti et al., 1999; Pournaras et al., 2005). Imipenemase (IMP) type MBLs are endemic in Japan (Osano et al., 1994), but have been detected worldwide (Limbago et al., 2011). Similar to *bla*_VIM_, genes encoding IMP variants (*bla*_IMP_) are carried on integrons and can be encoded on either chromosomes or are plasmid-mediated (Arakawa et al., 1995; Docquier et al., 2003). OXA carbapenemases are class D enzymes are characterized by their ability to hydrolyze cloxacillin or oxacillin (Bush and Jacoby, 2010). The plasmid encoded OXA-48 (*bla*_OXA-48_) is found in *K. pneumoniae* and confers a high level of resistance to imipenem (Poirel et al., 2004).

### Colistin resistance in *Klebsiella pneumoniae*

Colistin resistance in *K. pneumoniae* is commonly caused by mutations in the core genome, but transmissible resistance genes in the accessory genome are a grave concern. Colistin is among the polymyxin class of antibiotics, used to treat Gram-negative infections in the 1960s and 1970s. Their use was discontinued due to renal- and neurotoxicity (Jerke et al., 2016). However, the recent emergence of CRE has made it necessary to return to colistin as a drug of last resort. Colistin resistance in *K. pneumoniae* typically occurs through mutations in regulatory genes such as *mgrB* that regulate modification of bacterial lipid A, the target of polymyxin antibiotics, decreasing the ability of polymyxins to interact (Cannatelli et al., 2013; Jayol et al., 2014, 2015; Olaitan et al., 2014; Poirel et al., 2015; Wright et al., 2015). In 2015, plasmid-mediated resistance to colistin was discovered in an *E. coli* isolate in China (Liu et al., 2016), conferred by the *mcr-1* gene. This discovery heralds the potential for easily transmissible genes leading to pan-resistance. The prevalence of *mcr-1* in *K. pneumoniae* BSI isolates in China is rare, and found more often in *E. coli* (Quan et al., 2017). The first reported incidence of *mcr-1* in the United States occurred in 2016 in *E. coli* (McGann et al., 2016). In September of 2016 a pan-resistant isolate of *K. pneumoniae* was isolated (Chen et al., 2017), but colistin resistance in this isolate was not mediated by *mcr-1*.

### Colonization as a reservoir of antibiotic-resistant *K. pneumoniae* in hospitals

Similar to recent findings across *K. pneumoniae* in general, intestinal colonization by antibiotic-resistant *K. pneumoniae* can precede infection with the same strain (Selden et al., 1971). A 1971 prospective study determined that patients colonized with antibiotic-resistant *K. pneumoniae* after hospital admission developed infection with antibiotic-resistant *K. pneumoniae* at a higher percentage within 21 days compared to those who did not become intestinal carriers. They further compared serotypes of colonization isolates and subsequent infecting isolates, finding that 14 of 31 (45.2%) patients colonized after admission were infected with the same serotype, indicating that hospitalized patients are often colonized with the same isolate they become infected with. There were, however, two predominant serotypes circulating and causing infection [serotype 30 (*n* = 11) and serotype 63 (*n* = 8)], confounding the significance of these findings. Serotype 30 was also the most common serotype identified in both rectal (*n* = 23/34) and antibiotic-resistant infecting (*n* = 18/20) isolates from a previous collection within the same facility (Selden et al., 1971). In a cohort study of ESBL-KP colonized patients, 22% progressed to a positive clinical culture, with a median time of 2.7 days from a positive rectal swab (Harris et al., 2007). For CR-Kp, colonization can persist and spread silently for years in long-term care facilities (Viau et al., 2012). CR-Kp colonization can also trigger a clonal outbreak and newly colonized patients can develop fatal infections (Snitkin et al., 2012).

### Risk factors for infections caused by antibiotic-resistant *Klebsiella pneumoniae*

Risk factors for colonization and infection with antibiotic-resistant *K. pneumoniae* are often considered together, so the risk factors for infection specifically are unclear (Selden et al., 1971; Pollack et al., 1972; Asensio et al., 2000). Risk factors associated with ESBL colonization and infection include prior treatment with antibiotics, prolonged hospitalization, prolonged ICU stay, and mechanical ventilation (Jacoby, 1998; Lautenbach et al., 2001; Nathisuwan et al., 2001). Intestinal colonization with ESBL bacteria has also been associated with ESBL infection. Risk factors associated with carbapenem-resistant *K. pneumoniae* colonization and infection include prior antibiotic treatment, renal dysfunction, older age, surgical procedures, and ICU admission (Kofteridis et al., 2014; Jiao et al., 2015). As with endemic strains of *K. pneumoniae*, hospitalization seems to be a key factor associated with infection. Though antibiotic-resistant strains can infect an array of body sites similar to endemic strains, they frequently cause UTIs (Selden et al., 1971; Lautenbach et al., 2001). This may represent inoculation of the urinary tract with *K. pneumoniae* from the gastrointestinal tract across the perineum.

## Community-acquired hypervirulent strains

In the 1980s and 1990s, reports began to emerge from the Asian Pacific Rim detailing severe infections due to *K. pneumoniae* (Liu et al., 1986; Cheng et al., 1991; Wang et al., 1998). These infections were unique in that they were community-acquired (CA), a departure from the classic presentation of *K. pneumoniae* infections in hospitalized patients. Common infections due to these hypervirulent *K. pneumoniae* (hvKP) include pyogenic liver abscess (PLA); endophthalmitis, an infection inside the eye; meningitis; and bloodstream infections (Fang et al., 2007). Symptoms of PLA vary between individuals and are frequently non-specific. Diagnosis requires radiographic imaging (Johannsen et al., 2000). The incidence of PLA rose sharply in Taiwan, from 11.1 to 17.6 per 100,000 people from 1996 to 2004 (Fung et al., 2012). Approximately 3–11% of patients with PLA will go on to develop endophthalmitis (Sng et al., 2008; Sheu et al., 2011). Patient risk factors for severe infection with hvKP include being aged 55–60 years, male, and having diabetes mellitus (Lee et al., 2008; Siu et al., 2012; Shon et al., 2013). Throughout China there is a high prevalence of hvKP strains among *K. pneumoniae* isolates that cause infection (31–37.8%), though the rate varies based on geographic location within China (Liu et al., 2014; Zhang et al., 2016). Reported mortality rates (14–30 days) vary among patients in Asia who are infected with hvKP (4.5%-31%) (Wang et al., 1998; Ko et al., 2002; Liu et al., 2014). Since hvKP strains emerged from the Asian Pacific Rim and are overrepresented in individuals of Asian descent (Wang et al., 1998), it has also been suggested that infection with these strains is associated with an individual's ethnicity, or that it may be a geographically-specific pathogen, though it is unclear if this is a significant association (Shon et al., 2013). Alarmingly, these strains have begun to emerge worldwide, including in the United States (Lederman and Crum, 2005; Nadasy et al., 2007; Pastagia and Arumugam, 2008; Frazee et al., 2009; McCabe et al., 2010; Fierer et al., 2011). However, individuals from Western countries who become infected with hvKP are frequently either of Asian descent and/or have recently traveled to or been in contact with someone from an Asian country (Lederman and Crum, 2005; Keynan et al., 2007; Frazee et al., 2009; Gunnarsson et al., 2009; McCabe et al., 2010; Decré et al., 2011; Pomakova et al., 2012). Fortunately, hvKP isolates are typically highly susceptible to most antibiotics (Fang et al., 2007). Recently, however, an outbreak due to a carbapenem-resistant ST11 hvKP isolate occurred in China, heralding the potential for dual-risk isolates that both cause severe infections and are increasingly difficult to treat or are fatal due to antibiotic resistance (Gu et al., 2017).

### Hypervirulent *K. pneumoniae* possess unique virulence factors

The most striking aspect of hvKP isolates is their ability to cause severe infections in otherwise healthy patients. This severity is attributed to virulence factors encoded by the accessory genome. HvKP have a hypermucoviscous phenotype characterized by a positive “string” test: attempting to pick a colony with a loop results in a strand of bacteria that clings to the agar media. This phenotype has been determined to be conferred by two proteins: RmpA, which regulates capsule production (Hsu et al., 2011; Shon et al., 2013), and MagA, which is associated with the hypermucoviscous phenotype (Yu et al., 2006). Genes encoding RmpA and MagA are highly associated with hvKP, especially in Asia, and are considered virulence factors. Capsule types K1 and K2 are also highly associated with hvKP and may play a more important role in virulence than *rmpA* and *magA* (Yeh et al., 2007). Frequently, hvKP strains are K1 or K2 capsule type (Fung et al., 2002; Chung et al., 2007; Yu et al., 2008; Shon et al., 2013). They are also often sequence type 23 (ST23) a phylogroup strongly associated with the K1 capsule type (Liu et al., 2014). The siderophore Ybt has also been associated with hypervirulent strains (Holt et al., 2015), although it should be noted that Ybt is also found in many non-hypervirulent strains and is considered a general virulence factor in *K. pneumoniae*, as discussed above (Lawlor et al., 2007). Furthermore, the siderophore Aer has been distinguished as the most common siderophore secreted by hypervirulent *K. pneumoniae* (Russo et al., 2015).

Allantoin has been identified as a source of nitrogen in various bacterial species and as both a nitrogen source and a carbon source in *K. pneumoniae* (Cusa et al., 1999; Chou et al., 2004; Navone et al., 2014). Allantoin is a metabolic intermediate of purine degradation by various organisms including microbes (Vogels and Van der Drift, 1976). An allantoin utilization operon has been associated with hypervirulent *K. pneumoniae* strains that cause pyogenic liver abscesses (Chou et al., 2004). Deletion of a regulator gene in the operon corresponds to decreased virulence in a mouse model, indicating that the ability to use as a nitrogen source increases virulence in *K. pneumoniae* at certain sites of infection. Allantoin metabolism genes are variably encoded chromosomally in *K. pneumoniae*, and part of the accessory genome (Chou et al., 2004).

### Colonization as a reservoir for hvKP

Colonization appears to be a reservoir for hvKP that cause PLA and other severe infections. PFGE typing of liver and fecal isolates from patients with PLA demonstrates little or no differences within patients but discernable differences between patients. But each of these strains may have colonized many people, as a random sampling of fecal isolates from asymptomatic patients revealed some of the same PFGE types seen in PLA isolates (Fung et al., 2012). These findings suggest that, similar to opportunistic *K*. *pneumoniae*, hvKP infections are caused by endogenous colonizing strains.

If colonization is a potential reservoir for infection with hvKP strains, then understanding the community carriage rates is important. With that in mind, a recent study sought to identify the fecal carriage rate of K1 *K. pneumoniae* in healthy Koreans in order to identify this potential reservoir (Chung et al., 2012). They determined that 4.9% of individuals tested carry K1 *K. pneumoniae*. Of these, 94.7% were found to be ST23, which is strongly associated with PLA. The overall *K. pneumoniae* colonization rate was 21.1%. They further determined that the K1 carriage rate was higher in foreigners who lived in Korea compared to those of Korean descent who lived outside of Korea (24.1 vs. 5.6%, *P* = 0.024), suggesting that exposure to the Korean peninsula plays a role in K1 colonization. A similar study was undertaken where stool isolates from healthy Chinese adults living in various Asian countries were collected over 6 years and tested for the presence of *K. pneumoniae* with a K1/K2 serotype, both of which are associated with PLA (Lin et al., 2012). It was determined that 9.8% of *K. pneumoniae* isolates recovered were K1/K2. Rates were similar for Chinese living in all countries tested except Thailand and Vietnam. Strikingly, the overall *K. pneumoniae* colonization rate was also significantly higher than that seen in Western counties, with rates of >50% in cohorts from Taiwan and China. These findings establish that hvKP can be a significant proportion of *K. pneumoniae* colonization, and with high overall colonization rates, many people may be colonized with potentially deadly strains.

### Convergence of hypervirulence and carbapenemase accessory genes

The most obvious concern about *K. pneumoniae* and its flexible accessory genome is a dual-risk isolate, one that is both antibiotic-resistant and hypervirulent. With treatment options already limited, this result could be devastating. These isolates will cause severe disease but will be difficult or impossible to treat. Genomic analyses of strains worldwide have already detected the convergence of hypervirulence and carbapenemase genes in a number of isolates (Bialek-Davenet et al., 2014; Holt et al., 2015) As proof of the legitimacy of these concerns, a recent study identified a fatal outbreak of VAP due to a hypervirulent carbapenem-resistant *K. pneumoniae* ST11 isolate in China (Gu et al., 2017). These isolates had acquired portions of the hypervirulent virulence plasmid pLVPK, and contained several virulence factors associated with hypervirulent strains including genes encoding the siderophore Aer and the mucoid phenotype regulator gene *rmpA*. Alarmingly, no antibiotics were effective in treating the infections caused by these hv CRE *K. pneumoniae* strains. The ST11 strains identified in this study also included genes encoding the siderophore Ybt. Interestingly, Ybt is also found in a subset of ST11 across Asia (Holt et al., 2015). These CRE *K. pneumoniae* isolates already encode a critical virulence factor in Ybt and can acquire more through acquisition of hypervirulence plasmids.

## *Klebsiella* species vary in their accessory genome content

Improved molecular epidemiology and sequencing capabilities have recently demonstrated that isolates frequently identified as *K. pneumoniae* can be divided into three distinct *Klebsiella* species (Brisse and Verhoef, 2001; Brisse et al., 2004, 2014; Maatallah et al., 2014; Berry et al., 2015). Although initially distinguished by variations in their core genome, these species can also be separated by the content of their accessory genome (Holt et al., 2015). Phylogroup KpI represents ~80% of opportunistic isolates and is the species *K. pneumoniae* (*sensu stricto*). Phylogroup KpII is identified as *Klebsiella quasipneumoniae* and KpIII as *Klebsiella variicola*. As three distinct species, these groups may vary in their epidemiology of colonization and infection.

Not much is known about the least frequently isolated of the three Kp phylogroups, *K. quasipneumoniae*. Approximately 94% of *K. quasipneumoniae* isolates have been recovered from humans, with over 50% of human isolates being associated with intestinal carriage rather than infection (compared to 24 and 39% intestinal carriage in KpI and KpIII, respectively; Holt et al., 2015). The most common sites of *K. quasipneumoniae* infections are the urinary and respiratory tracts, and some isolates are ESBL-producers (Holt et al., 2015).

*Klebsiella variicola* was proposed as a new, distinct species based on both genetic and biochemical differences from *K. pneumoniae* (Rosenblueth et al., 2004; Brisse et al., 2014). A consistent trait of *K. variicola* is the ability of this species to fix nitrogen, which explains their endophytic relationship with several plants such as maize, banana, sugarcane, and wheat. Genes responsible for nitrogen fixation, such as those in the *nif* operon, can be considered part of the core genome of *K. variicola*, but can also be found in related *Klebsiella* species as part of the inter-species accessory genome (Fouts et al., 2008). While colonization of plants is common, colonization rates in humans is unknown. Though this species is associated with environmental sources, the clinical importance of *K. variicola* is becoming more apparent. In fact, a recent study determined that patients with bloodstream infections due to *K. variicola* demonstrate a higher 30-day mortality rate (29.4%) compared to *K. pneumoniae* (13.5%) and *K. quasipneumoniae* (11.1%) species (Maatallah et al., 2014). This increased mortality was not due to any known virulence factors and was significant after controlling for patient co-morbidities, suggesting that *K. variicola* harbors yet undiscovered and potentially clinically relevant genes.

A comparison of the antimicrobial resistance patterns of clinical isolates of the three Kp phylogroups (*n* = 420) to 10 antimicrobial agents showed that *K. pneumoniae* (KpI) has the highest resistance levels, *K. quasipneumoniae* (KpII) has intermediate resistance levels, and *K. variicola* (KpIII) has the lowest resistance, with KpI having a resistance rate of two- to three-fold higher than KpIII (Brisse et al., 2004). A more recent study of isolates from the three phylogroups collected worldwide (*n* = 328) determined that ~50% of KpII isolates display an ESBL phenotype (Holt et al., 2015).

Since it is only relatively recently that these species have been differentiated, there is a paucity of information in the literature regarding colonization rates and virulence factors in *K. quasipneumoniae* and *K. variicola*. Along with *K. pneumoniae*, these three distinct but related species vary in their epidemiology of infection and antimicrobial resistance, likely due to the variation in genes each species possesses, indicating that exploration of the *Klebsiella* accessory genome is imperative for better understanding the nuances of how these bacteria cause disease. Indeed, genetic exchange across these three species can occur (Holt et al., 2015; Long et al., 2017a).

## Summary and gaps in knowledge

Though classically considered an opportunistic, hospital-acquired pathogen that infects only immunocompromised hosts, two additional types of *K. pneumoniae* have emerged: carbapenem-resistant and hypervirulent. Across these three types, intestinal colonization rates are significant and serve as a reservoir for isolates capable of causing infection. For hospital-onset infections, the association between colonization and subsequent infection is established and strong. For hvKP and CR-KP the association between colonization and subsequent infection is unclear. And for all types of *K. pneumoniae*, the risk factors for progression to infection in colonized patients are poorly understood. Understanding colonization and infection as two distinct stages with potentially varying risk factors will further aid in understanding the pathogenesis of *K. pneumoniae*.

**Figure 1:**
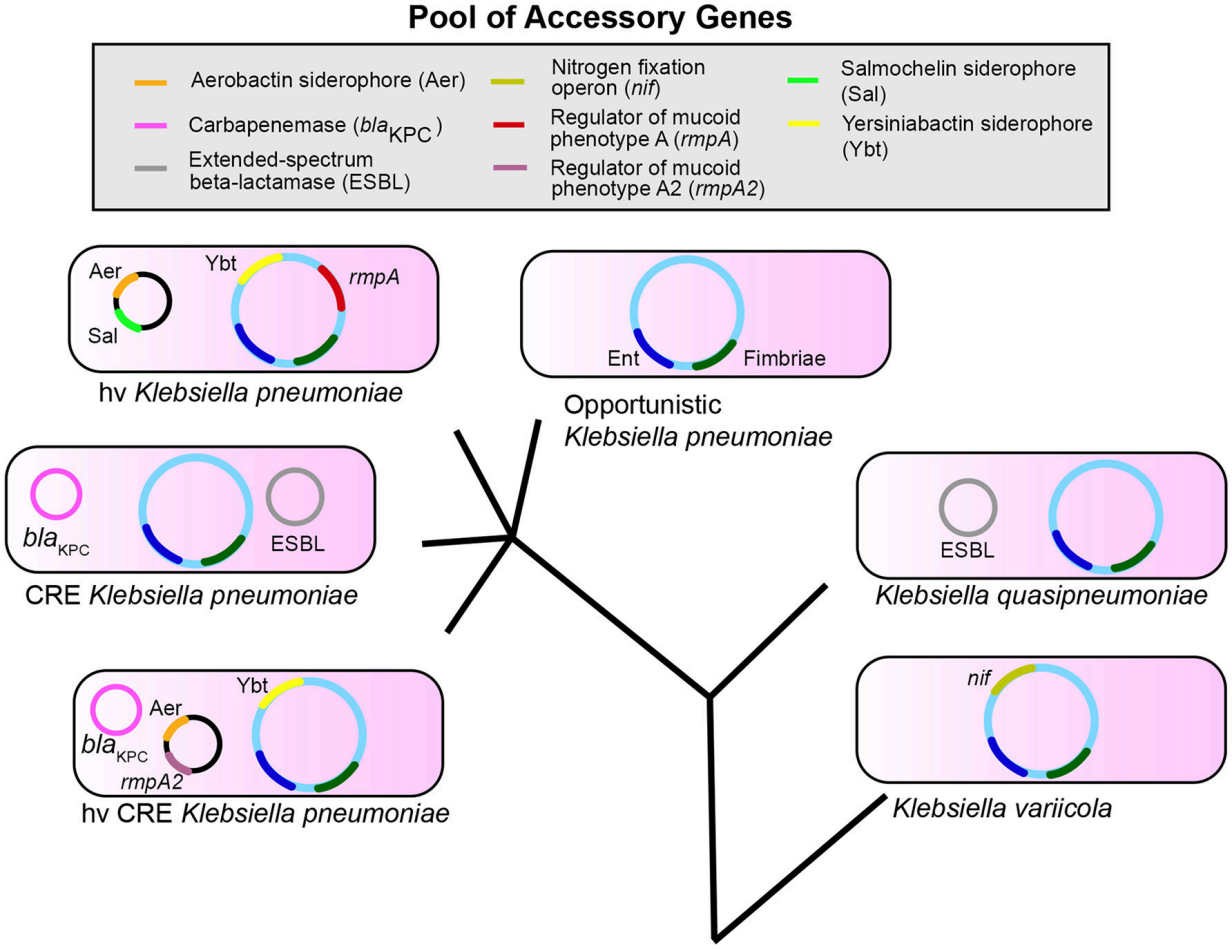
*Klebsiella pneumoniae, variicola*, and *quasipneumoniae* are three species that share a pool of accessory genes. The combination of genes in the accessory genome differs between species and pathotypes within *K. pneumoniae* such as opportunistic, carbapenem-resistant (CRE), and hypervirulent (hv) strains. These accessory genes can also combine to form new pathotypes (hvCRE) and can be shared across species. Enterobactin (Ent, blue) and Fimbriae (dark green) represent conserved genes; accessory genes are shown as examples and are not a definitive list. The evolutionary tree is not drawn to scale.

The accessory genome is likely critical in determining the differences in infection risk and outcomes of endemic, antibiotic-resistant, and hypervirulent *K. pneumoniae*. Several *K. pneumoniae* virulence factors identified to date are based on association with hypervirulent phenotypes, such as PLA. Furthermore, most studies involving *K. pneumoniae* tend to focus on one clonal group, such as carbapenem-resistant or hypervirulent isolates. While understanding of pathogenesis for each distinct type is important, a broad understanding of how *K. pneumoniae* causes the common infections of pneumonia, bacteremia, and UTIs could better aid in targeting common factors for diagnosis and treatment. Finally, it is only recently that we have begun to recognize that ~20% of nosocomial isolates identified as *K. pneumoniae* are actually *K. variicola* and *K. quasipneumoniae*. These species may have distinct epidemiological and resistance profiles based on the composition of their accessory genome. However, it is becoming evident that there is a reservoir of genes that are exchanged and assembled to create strains with varying infectious and antibiotic-resistant potential (Figure 1). Therefore, it is increasingly clear that *Klebsiella* can assemble a large accessory genome from a larger pool of available genes to determine their ability to colonize, infect, and resist antibiotics in humans.

## Author contributions

RM and MB: conceived design of the review, drafted, and critically revised the work, and provided final approval for publication.

### Conflict of interest statement

The authors declare that the research was conducted in the absence of any commercial or financial relationships that could be construed as a potential conflict of interest.
